# Chitin, Chitosan, and Glycated Chitosan Regulate Immune Responses: The Novel Adjuvants for Cancer Vaccine

**DOI:** 10.1155/2013/387023

**Published:** 2013-03-04

**Authors:** Xiaosong Li, Min Min, Nan Du, Ying Gu, Tomas Hode, Mark Naylor, Dianjun Chen, Robert E. Nordquist, Wei R. Chen

**Affiliations:** ^1^Department of Oncology, The First Affiliated Hospital of Chinese PLA General Hospital, Beijing 100048, China; ^2^Department of Gastroenterology, Affiliated Hospital of Academy of Military Medical Sciences, Beijing 100071, China; ^3^Department of Laser Medicine, Chinese PLA General Hospital, Beijing 100853, China; ^4^ImmunoPhotonics Inc., Columbia, MO 65211, USA; ^5^Dermatology Associates of San Antonio, San Antonio, TX 78233, USA; ^6^Department of Engineering and Physics, University of Central Oklahoma, Edmond, OK 73034, USA

## Abstract

With the development of cancer immunotherapy, cancer vaccine has become a novel modality for cancer treatment, and the important role of adjuvant has been realized recently. Chitin, chitosan, and their derivatives have shown their advantages as adjuvants for cancer vaccine. In this paper, the adjuvant properties of chitin and chitosan were discussed, and some detailed information about glycated chitosan and chitosan nanoparticles was also presented to illustrate the trend for future development.

## 1. Adjuvant

Activating the immune system for therapeutic benefit has long been a goal in immunology, especially in cancer treatment [1–3]. However, for the development of therapeutic vaccines to treat cancer patients, there are still some obstacles to overcome. The tumor antigens are usually self-derived and are, therefore, poorly immunogenic. Tumors develop escape mechanisms to avoid the immune system, such as tumor editing, low or nonexpression of MHC class I molecules or secretion of suppressive cytokines. Moreover, the immune systems of cancer patients are often compromised, leading to impaired mechanisms of antigen presentation, nonresponsiveness of activated T cells, and enhanced inhibition of self-reactivity by regulatory T cells [4–6]. With the deeper understanding of the crosstalk between the host immune system and antigens, the important role of adjuvant in cancer vaccine has been proposed [7]. 

### 1.1. Concept and Classification of Adjuvant

Adjuvants are compounds that stimulate the immune system and increase the response to a vaccine, without having any specific antigenic effect on their own [8]. Based on their principal mechanisms of action, adjuvants can be generally divided into two classes [9, 10]: (1) vaccine delivery systems, such as mineral salts, emulsions, liposomes, and virosomes [11–13]; (2) immunostimulatory adjuvants, including toll-like receptor (TLR) agonists (e.g., monophosphoryl lipid A), saponins, and cytokines [14–16]. 

### 1.2. Mechanism of Adjuvant

Adjuvants exert their effect by several different mechanisms. Vaccine delivery systems can help present antigens to the immune system of the host in a more efficient way and control the release and storage of the antigens. Immunostimulatory adjuvants affect the immune system and increase the immune responses to antigens. Cytokine production is increased by the activation of MHC molecules, costimulatory signals, or through related intracellular signaling pathways, leading to the enhanced specific immune response against antigens. 

### 1.3. Commonly Used Adjuvants

It has been over 80 years since the discovery of the adjuvant activity of aluminum compounds [17]. Although hundreds of adjuvant candidates have been tested both preclinically and clinically, most of them failed to be approved for routine usage [18]. The most common adjuvants for human use today are still aluminum hydroxide and aluminum phosphate. However, because of its aluminum-related macrophagic myofasciitis, the application of aluminum hydroxide has not been widely used.

During the last decades, much progress has been made on the development of immunoadjuvants. Several novel adjuvants are licensed for human use in different countries, including aluminum salts, squalene-oil-water emulsion (MF-59), monophosphoryl lipid (MPL) A and virosomes [19, 20]. However, it is still in great need to invent novel adjuvant for clinical practice. An “ideal” adjuvant would elicit a persistent, high-affinity immune response to an antigen while being nontoxic, biodegradable, nonimmunogenic and chemically defined for reproducible manufacture [21, 22]. Development of safe, novel adjuvants is necessary for immunotherapy [23, 24].

## 2. Chitin and Chitosan

### 2.1. Chitin

**Figure 1:**
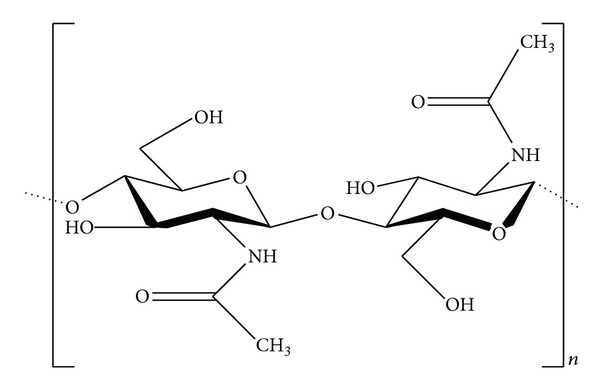
Chemical structure of the chitin molecule.

Chitin is a long-chain polymer of N-acetylglucosamine, a derivative of glucose (chemical structure shown in Figure 1) [25]. It is the second most abundant polysaccharide in nature and is commonly found in lower organisms. It is the main component of the cell walls of fungi, the exoskeletons of arthropods such as crustaceans (e.g., crabs, lobsters, and shrimps) and insects, the radulas of mollusks, and the beaks of cephalopods, including squids and octopuses [26]. 

### 2.2. Chitosan

**Figure 2:**
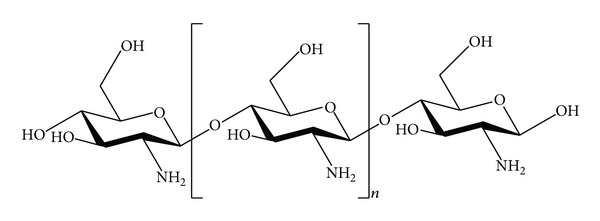
Chemical structure of the chitosan molecule.

Chitosan is a nontoxic, biocompatible, biodegradable, natural polysaccharide, which is converted from chitin by deacetylation (chemical structure shown in Figure 2) [27]. A common method for the synthesis of chitosan is the deacetylation of chitin using sodium hydroxide in excess as a reagent and water as a solvent [28]. The process causes changes in molecular weight and a degree of deacetylation of the product and degradation of nutritionally valuable proteins. The molecular weight of chitosan is between 3800 and 20,000 Daltons. The degree of deacetylation (%DD) ranges from 60% to 100%. 

### 2.3. Application of Chitin and Chitosan

Their biodegradability, biocompatibility, and nontoxicity provide chitin and chitosan with huge potential for future development. Chitin and chitosan are widely applied in chemistry, biotechnology, agriculture, veterinary, dentistry, food processing, environmental protection, and medicine. Chitosan can be easily processed in diverse forms, such as films, threads, tablets, membranes, and microparticles/nanoparticles, allowing the design of a variety of medical and pharmacological devices adaptable to end purposes [29, 30].

Although the mechanism of interaction between chitosan and fat is not very well understood and has not been proved clinically yet, chitosan has been used as an effective complement to help lose weight during diet period or to stabilise one's weight [31, 32].

Since they are biocompatible, biodegradable, mucoadhesive, and nontoxic, with antimicrobial, antiviral, and adjuvant properties, chitin and chitosan have been widely applied in medicine and pharmacy [33]. They promote the continuous movement for the development of safe and effective drug delivery systems because of their unique physicochemical and biological characteristics. Combined with drugs such as doxorubicin, paclitaxel, docetaxel, and norcantharidin, chitin and chitosan are used as drug carriers. With their low molecular weight, chitin and chitosan are useful carriers for molecular drugs requiring targeted delivery [34–36]. Their biodegradability makes them dissolve with time when used in wound healing. Moreover, chitin has some unusual properties that accelerate healing of wounds in humans [37, 38].

## 3. Adjuvant Properties of Chitin and Chitosan

Besides all application mentioned earlier, chitin and chitosan are important adjuvants for immunotherapy. This immunostimulating activity along with the structural similarities between chitin derivatives and glucans, an adjuvant class of natural polysaccharides, led many researchers to investigate the adjuvant properties of chitin and chitosan. 

The nonspecific antiviral and antitumor activities of chitin and chitosan were described about three decades ago. In the 1980s, the adjuvant effect of chitin and chitosan was first demonstrated by Suzuki et al. [39]. Nishimura et al. proved the immunological activity of chitin derivatives and further compared their effect as adjuvant [40–42]. They found out the following: (1) For the activation of peritoneal macrophages *in vivo*, 30% deacetylated chitin (DA-chitin), 70% carboxymethyl-chitin (CM-chitin) induced cytotoxic macrophages most effectively. Chitosan, hydroxyethyl-chitin, dihydroxypropyl-chitin (DHP-chitin), and DHP-chitosan had moderate activities. Phosphorylated-, sulphonated-, or acetyl-chitin, however, was less effective. (2) For the suppression of Meth-A tumor growth in BALB/c mice, 70% DA-chitin and DHP-chitosan were most active, and 30% DA-chitin had a moderate effect. (3) For the stimulation of nonspecific host resistance against *Escherichia coli* infection, 30% and 70% DA-chitin were both effective. 

Because of its mucoadhesive properties, chitin and chitosan are widely applied for mucosal routes of administration, that is, oral, nasal, and ocular mucosa, which are noninvasive routes. The enhancement of adaptive immune responses to several antigens has been proved [43]. Recent clinical studies have confirmed that chitosan is a promising adjuvant platform for intranasal vaccination [44, 45]. The mechanisms of vaccine enhancement by chitosan through mucosal administration are believed to be due to both retention of vaccine in the nasal passages via mucoadhesion and opening of endothelial cell junctions for paracellular transport of vaccine [46].

 Several other routes for the administration of chitin and chitosan were proposed, such as intraperitoneal (i.p.), intravenous (i.v.), subcutaneous (s.c.), and intratumoral (i.t.) administrations [47–50]. Given by intravenous administration, significant priming effects of chitin particles in alveolar macrophages and NK cells in mice were observed by Shibata et al. [51]. Phagocytosable small-sized chitin particles activated alveolar macrophages to express cytokines such as IL-12, tumor necrosis factor-*α* (TNF*α*), and IL-18, leading to INF-*γ* production mainly by NK cells. They further demonstrated that the cytokine production was through mannose receptor-mediated phagocytosis [52].

Tokura et al. proved that macrophages were activated to various extents by chitin derivatives, and 30% DA-chitin and chitin sulfate stimulate the production of circulating antibodies [53]. Accumulation of carboxymethyl chitin took place in granulocytes and macrophages. They also found out that intraperitoneal injection of N-acetylchitohexaose inhibited the growth of tumor cells and pathogens.

Chitin has complex and size-dependent effects on innate and adaptive immune responses, which include the ability to recruit and activate innate immune cells and induce cytokine and chemokine production [54]. The involved cell surface receptors include macrophage mannose receptor, toll-like receptor 2 (TLR-2), C-type lectin receptor Dectin-1, and leukotriene B4 receptor (BLT1) [55].

Da Silva et al. demonstrated in their experiment that chitin particles using intraperitoneal injection were potent multifaceted adjuvants that induced adaptive Th2, Th1, and Th17 immune responses [56]. TLR-2, MyD88, and IL-17A have been proved to play important roles in the adjuvant properties of chitin and chitosan [56].

The immune response induced by chitin and chitosan is determined by the existence of antigen. Marcinkiewicz et al. found that i.p. administration of chitosan alone enhanced humoral responses but not cell-mediated immune responses in mice [57]. Subcutaneous administrations of chitosan without antigen were found to be ineffective. In other studies, Seferian and Martinez found that chitosan particles, formulated in an emulsion with antigen, squalene, and Pluronic L121, gave a prolonged, high antigen-specific antibody titer and sensitized animals for antigen-specific DTH responses following an i.p. injection [58]. 

It is believed that chitosan enhances both humoral and cell-mediated immune responses. Zaharoff et al. explored chitosan solution as an adjuvant for subcutaneous vaccination of mice with *β*-galactosidase, a model protein antigen [59]. They found that chitosan enhanced antigen-specific antibody titers over fivefold and antigen-specific splenic CD4+ proliferation over sixfold. Strong increases in antibody titers together with robust delayed-type hypersensitivity (DTH) responses revealed that chitosan induced both humoral and cell-mediated immune responses. They also compared chitosan with traditional vaccine adjuvants and proved that chitosan was equipotent to incomplete Freund's adjuvant (IFA) and superior to aluminum hydroxide. It was hypothesized that a viscous chitosan solution, when administered subcutaneously, would not only provide immune stimulation as previously described but also act as an antigen depot [41]. 

Recombinant granulocyte-macrophage colony-stimulating factor (rGM-CSF) expedites neutrophil recovery in cancer patients receiving chemotherapy. When coformulated with chitosan, local rGM-CSF retention at a subcutaneous injection site in mice was increased for up to 9 days [60]. In contrast, when delivered in a saline vehicle, rGM-CSF was undetectable in 12–24 h [60]. This indicated that chitosan helped control the dissemination of rGM-CSF. IL-12 is a potent antitumor cytokine that exhibits significant clinical toxicities after systemic administration. When given in tumor-bearing mice, intratumoral administration of IL-12 coformulated with chitosan (chitosan/IL-12) could help limit its systemic toxicity, by increasing local retention of IL-12 in the tumor microenvironment (chitosan/IL-12 versus IL-12 alone: 5-6 days versus 1-2 days) [61]. Moreover, the enhancement of antitumor efficacy of chitosan/IL-12 and chitosan/rGM-CSF was observed by Zaharoff et al. [60, 61].

## 4. Chitosan Derivatives: Glycated Chitosan

Many chitosan derivatives, such as trimethyl chitosan, Zwitterionic chitosan, and glycated chitosan, have been invented recently [62–65]. With the development of nanotechnology, chitosan have shown its unique advantages when combined with nanoparticles. Improved biodegradability, immunological activity, and high viscosity can be achieved by modification of chitosan, which makes them excellent candidates as adjuvants. 

Chitosan is soluble in diluted acids but is relatively insoluble in water [66, 67]. The poor solubility of chitosan poses limitations for its biomedical applications. Especially in the areas of immunology, an aqueous solution is essential for its use as an immunostimulant in clinical applications. By attaching galactose molecules to the chitosan molecules, a new water-soluble compound, glycated chitosan (GC), was formed [68, 69]. 

GC was synthesized by incubating an aqueous suspension of chitosan with a threefold excess of galactose and subsequent stabilization by borohydride reduction of the Schiff bases and Amadori products [70]. GC is a synthesized nontoxic biodegradable product, which is the drug component of inCVAX (Immunophotonics Inc., Columbia, MO), which has been also referred to as laser immunotherapy (LIT). LIT uses a combination of local laser irradiation and local administration of GC at either primary or metastatic tumor sites [71]. Employing synergistic photothermal effect and immunological effect, laser immunotherapy has been proved to be a promising treatment modality for metastatic cancers [72]. Through intratumoral administration, GC is usually given after laser irradiation of the tumor lesions. GC exerts its effect as immunoadjuvant for treatment of metastatic cancers [73]. 

During laser irradiation, the temperature in the treated lesion could reach up to 60–70°C [74]. The irradiated tumor cells would swell and break into pieces by thermal effect, creating antigen sources to stimulate the host immune system to generate tumor-specific immune responses [3, 75]. These antigens include tumor-associated antigens, thermally induced heat shock proteins (HSPs), and a large amount of self-antigens. Antigen presenting cells (APCs), particularly dendritic cells (DCs), can capture these antigens and migrate to lymph nodes, where they present these antigens to T cells, thus activating cytotoxic T-lymphocytes (CTLs) [76–78]. 

Heat shock proteins, including calreticulin (CRT), HSP70, and gp96, have been shown to act as potent immunoadjuvants to enhance antigen-specific tumor immunity [79–81]. HSPs can promote the cross-presentation of HSP-bound peptide antigens to major histocompatibility complex (MHC) class I molecules in DCs, leading to induction of antigen-specific CTL [82, 83]. It is proposed that GC exerts its effect through the pathway similar to that of chitin and chitosan [84, 85]. It has been also observed that TNF-*α* and INF*γ* secretion was increased when mouse macrophages were incubated with GC, which was also clearly dose dependent [68, 86]. 

Animal experiments showed that LIT using GC could have a high potential for the treatment of metastatic tumors by inducing a tumor-specific, long-lasting immunity [87]. Primary tumors and metastases began to regress about several weeks after LIT in successfully treated rats. Furthermore, these successfully treated tumor-bearing rats could resist repeated challenges with the tumors of same type and also escalated tumor doses. Passive adoptive transfer was further performed using splenocytes as immune cells and the spleen cells harvested from successfully treated tumor-bearing rats provided 100% immunity in the naive recipients [88]. The passively protected first cohort rats were immune to tumor challenge with an increased tumor dose; their splenocytes also prevented the establishment of tumor in the second cohort of naive recipient rats. In another experiment, Balb/C mice bearing EMT6 tumor achieved long-term tumor-specific response, which helps the successfully treated mice resist repeated tumor rechallenge [89]. 

A clinical trial for the treatment of late-stage breast cancer patients, who have failed other available modalities, has been carried out recently [72]. It has been proved that LIT is well tolerated, which allows repeated LIT applications. The most common adverse effects were rash and pruritus at the treatment sites. No grade 4 toxicity was observed. Preliminary results showed that LIT was capable of reducing the size of treated primary breast tumors and untreated metastases in the lungs [72]. 

GC has shown several advantages when working as an immunoadjuvant in LIT [79]. Local application of GC induces low toxicity, which makes it suitable for late-stage cancer patients who cannot tolerate chemotherapy and radiation therapy [72, 87]. It also can be applied repeatedly without dose limiting effect. By combination with laser irradiation, significant systemic antitumor immunity is generated [85, 90]. Improved survival time for metastatic cancer patients is observed in clinical trial [72]. 

## 5. Chitosan Nanoparticles

Improved biodistribution, increased specificity and sensitivity, and reduced pharmacological toxicity are achieved by combining chitosan with nanoparticles, making them perfect polymeric platforms for the development of new pharmacological and therapeutic drug release systems [90]. Chitosan nanoparticles (CNPs) can be administrated through noninvasive routes such as oral, nasal, pulmonary, and ocular routes [91]. Moreover, CNPs have been proposed as nonviral vectors in gene therapy and have shown their adjuvant effect in vaccines [92]. CNPs are also used for chemotherapy drug delivery by combining them with monoclonal antibody with reduction of drug side effects in some specific cancer patients [93]. 

CNP may be a safe and efficacious adjuvant candidate suitable for therapeutic vaccines. Experimental results have proved that CNP had a strong potential to increase both cellular and humoral immune responses and elicited a balanced Th1/Th2 response. Wen et al. investigated the promoted immune response to ovalbumin (OVA) in mice by CNP and its toxicity [94]. The mice were immunized subcutaneously with OVA alone or with OVA dissolved in saline containing Quil A, chitosan, or CNP. CNP did not cause any cell mortality or side effects. They observed that the serum OVA-specific IgG, IgG1, IgG2a, and IgG2b antibody titers and Con A-, LPS-, and OVA-induced splenocyte proliferation were significantly enhanced by CNP as compared with OVA and chitosan groups. CNP remarkably increased the killing activities of NK cells activity [95]. CNP also significantly promoted the production of Th1 (IL-2 and IFN-*γ*) and Th2 (IL-10) cytokines and upregulated the mRNA expression of IL-2, IFN-*γ*, and IL-10 cytokines in splenocytes from the immunized mice compared with OVA and chitosan groups [95]. 

Wu et al. showed that humoral and cellular immunities were significantly enhanced in immunized mice, which resisted the infection of *E. coli* and survived, while the control mice manifested evident symptoms and lesions of infection [95]. Their results showed that the inoculation with CpG-CNP significantly raised the content of IgG, IgM, and IgA in the sera of immunized mice. Increased number of white blood cells and lymphocytes and elevated levels of IL-2, IL-4, and IL-6 were also observed in the mice of CpG-CNP group. It indicates that CpG-CNP can be utilized as an effective adjuvant to improve the immunoprotection and resistance of porcine against infectious disease [96].

Modified chitosans can be used for the wide range of biomedical applications including the interaction and intracellular delivery of genetic materials [97]. Its unique properties help form a complex with siRNA [98]. Chitosan-based nanoparticles have been considered as a potential carrier for various gene delivery applications, which indicate its promising perspectives in gene therapy. 

## 6. Conclusions

Chitin and chitosan derivatives are well-tolerated, effective adjuvants with considerable potential for clinical practice. The immune response induced by chitin and chitosan is determined by the existence of antigen. It is believed that chitosan enhances both humoral and cell-mediated immune responses when vaccinated with antigen. 

Glycated chitosan apparently possesses improved properties over chitin and chitosan for immunological stimulation in clinical studies. However, more work needs to be done to dissect the signaling pathways and the effects on host immune cells, which will help understand the molecular mechanisms involved in the induction of adaptive immune responses induced by GC.

Combining with laser photothermal interaction and possibly other treatment modalities, GC's effects can be significantly enhanced when treating late-stage, metastatic cancers, as shown in preclinical and clinical studies. Further investigations are needed to understand the mechanisms of LIT with GC and to explore optimal treatment protocols for future clinical applications.
